# Walking stability prediction for pedestrians using gait energy images and hybrid deep and few-shot learning models

**DOI:** 10.1038/s41598-026-61650-z

**Published:** 2026-07-17

**Authors:** Mahmoud Taha, Ahmed Fares, Hirozumi Yamaguchi, Ahmed B. Zaky

**Affiliations:** 1 Department of Computer Science and Engineering, Egypt-Japan University of Science and Technology, Alexandria, Egypt; 2 Computer Science and Information Technology, Egypt-Japan University of Science and Technology, Alexandria, Egypt; 3 Graduate School of Information Science and Technology, Osaka University, Osaka, Japan; 4 Faculty of Engineering at Shoubra, Benha University, Cairo, Egypt

**Keywords:** Unstable walking recognition, Gait energy image, Human action analysis, Gait analysis, Few-shot learning, Deep learning, Support vector machines, Computational biology and bioinformatics, Engineering, Mathematics and computing

## Abstract

The prediction and recognition of unstable human walking patterns are of high importance for active video surveillance, smart environments, and assistive healthcare, particularly for fall detection in the elderly. This research investigates the utility of Gait Energy Images (GEIs) combined with deep learning, vision transformers, and few-shot learning architectures to enhance the classification of stable and unstable pedestrian walking patterns. We evaluate and compare twelve methodologies: six classical or feature-based machine learning models (Linear SVM, HOG + SVM, LBP + RBF-SVM, Random Forest, XGBoost, and an adapted GaitSet baseline), three deep learning models (MobileNet, Vision Transformer (ViT), and YOLOv8-cls), and three episodic few-shot learning techniques (Prototypical, Matching, and Relation Networks) under data-scarcity regimes. To facilitate this evaluation, we introduce the Unstable and Stable Walking Pedestrian (USWP) dataset, constructed by fusing and harmonizing sequences from seven public action recognition databases, containing 3250 unique GEIs with a subject-independent evaluation protocol to prevent identity-based domain leakage. Our experiments demonstrate that the YOLOv8-cls model achieves an overall accuracy of 96.92% (97.14% on Loss-of-Balance anomalies and 94.67% on Active Motion anomalies), significantly outperforming the conventional Linear SVM baseline (75.38%) and MobileNet (91.08%). Conversely, Relation Networks exhibit lower few-shot performance (71.08%) due to optimization complexities in learning similarity metrics from sparse data. Leave-One-Dataset-Out (LODO) cross-validation reveals an average generalization accuracy of 83.28%, indicating significant domain bias across source databases and underscoring that within-dataset evaluations overestimate real-world generalization. Computational complexity analysis shows that MobileNet provides an optimal trade-off for real-time edge deployment (4.2 ms latency), while preprocessing ablation studies demonstrate that integrating the Segment Anything Model (SAM) with MediaPipe-derived Regions of Interest (ROIs) yields a 12.50% absolute improvement in accuracy by eliminating background noise.

## Introduction

With the rapid advancements in the field of computer vision, pedestrian action recognition has emerged as a cornerstone task in security, video surveillance, video indexing, and human-computer interaction^1,2^. Within this domain, predicting walking stability and detecting gait abnormalities are critical for proactive healthcare, public safety, and smart environments. Sykes^3^ highlights the clinical importance of fall detection in older adults, a population highly vulnerable to falls due to age-related physical and cognitive decline. Falls not only represent major health threats but also impose severe economic burdens on healthcare systems worldwide. Existing fall and gait instability detection methodologies are broadly categorized into wearable or mobile device-based, pressure sensor-based, vision-based, and human pose estimation-based approaches. While wearable and pressure-based sensors require active user cooperation or expensive environmental modifications, vision-based systems offer a non-invasive, contact-free, and scalable solution that is highly suited for public spaces and assistive healthcare environments.

**Fig. 1:**
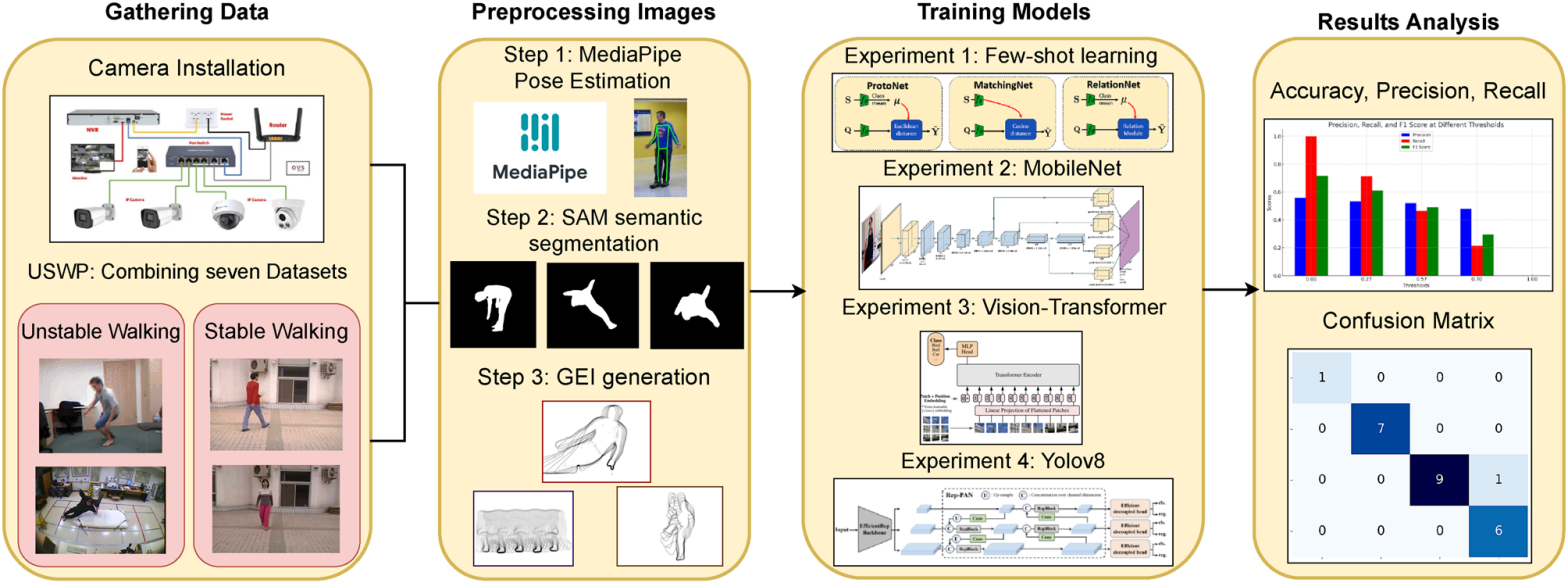
The proposed system framework for stable and unstable walking prediction. (1) Input sequence preprocessing utilizing MediaPipe Pose landmark extraction and SAM semantic segmentation. (2) Alignment and average projection to generate Gait Energy Images (GEIs). (3) Model evaluation using deep learning (MobileNet, ViT, YOLOv8), conventional machine learning (SVM), and episodic few-shot learning (Prototypical, Matching, and Relation Networks).

To address these challenges, we propose a comprehensive walking stability prediction framework, as illustrated in Fig. 1. A major hurdle in vision-based gait anomaly detection is the conceptual definition of “unstable walking.” Prior literature often fails to differentiate between gait instabilities caused by physical distress and general non-walking action anomalies. To resolve this ambiguity, we establish a rigorous classification of gait instability, categorizing anomalies into two distinct subgroups: (1) Loss-of-Balance (LoB) anomalies, which encompass unintentional gait disruptions such as lateral staggers, path deviations, swaying, and physical collapse (e.g., simulated drunkenness or falling); and (2) Active Motion (AM) anomalies, which denote intentional, dynamic, non-periodic bodily movements that interrupt regular locomotion (e.g., boxing, kicking, skipping, and swinging). Treating active motions as a separate subgroup allows us to evaluate whether a model is learning genuine gait instability features or simply acting as a coarse action classifier distinguishing walking from non-walking activities.

To benchmark these models, we construct the Unstable and Stable Walking Pedestrian (USWP) dataset by merging and harmonizing silhouettes from seven public action recognition databases. Preprocessing is applied to enhance classification; specifically, Segment Anything Model (SAM) semantic segmentation is combined with MediaPipe pose estimation to automate region-of-interest (ROI) detection and silhouette extraction, which are then compiled into Gait Energy Images (GEIs). We evaluate standard deep learning architectures, classical and feature-based machine learning baselines, and episodic few-shot networks.

In unconstrained urban environments, vision-based pedestrian analysis is further exacerbated by camera viewpoint variations, occlusions, dynamic backgrounds, and illumination changes. Zereen et al.^4^ proposed a real-time fall detection and unstable motion video system using Jetson TX2 modules and LSTMs, achieving 91.6% accuracy for daily activities. However, temporal modeling on raw video frames incurs substantial computational overhead. GEIs compress the spatial and temporal gait dynamics into a single 2D image, reducing memory footprints and enabling real-time edge processing^5^.

The principal contributions of this work are summarized as follows:

1. We introduce the Unstable and Stable Walking Pedestrian (USWP) dataset, a large-scale, unified dataset compiled from seven public repositories, providing a diverse benchmark for walking stability under various viewpoints and conditions.
2. We propose a fully automated preprocessing pipeline combining MediaPipe Pose estimation and the Segment Anything Model (SAM) to isolate clean, noise-free silhouettes and generate high-quality GEIs, mitigating manual seeding bias.
3. We conduct a comprehensive benchmark comparing twelve architectures, including six classical or feature-based baselines (Linear SVM, HOG+SVM, LBP+RBF-SVM, Random Forest, XGBoost, and an adapted GaitSet baseline), three standard deep learning models (MobileNet, Vision Transformers (ViT), YOLOv8 classification), and three episodic few-shot learning paradigms.
4. We perform a Leave-One-Dataset-Out (LODO) cross-dataset validation and subgroup performance analysis to address domain bias and clarify the performance difference between loss-of-balance anomalies and active motion anomalies.
5. We perform extensive ablation and computational complexity studies, reporting FLOPs, parameter counts, and inference latency, and include rigorous statistical significance tests (McNemar test with Bonferroni correction) and 95% confidence intervals across 5 repeated runs.

## Related work

### Silhouette-based gait recognition

Gait analysis has traditionally focused on human identification. Han and Bhanu^6^ introduced the Gait Energy Image (GEI), which represents spatio-temporal gait characteristics by averaging binary silhouette frames over a gait cycle. GEIs preserve both shape and stride dynamics at a low dimensional cost. Subsequent researchers improved GEI robustness against lighting and clothing variations using Gait Entropy Images^7^ and cross-view transformation models^8,9^. Recently, set-based representations like GaitSet^10,11^ have been proposed to model gait from independent silhouettes. However, for walking stability prediction, GEIs remain highly descriptive due to their ability to capture lateral posture swaying and sudden motion deviations in a single compact image.

### Pedestrian action and stability analysis

Pedestrian motion prediction and stability analysis represent vital components of smart cities and assistive technologies^12^. Several deep learning architectures have been applied to video-based activity recognition. For instance, Sun et al.^13^ developed a multi-channel fusion network utilizing skeletal joint images, while Zhao et al.^14^ integrated gait and personality features for depression assessment in older adults. Furthermore, infusing convolutional neural networks with joint node images has demonstrated excellent classification results on daily activities^15^.

Researchers have explored optimal transport bridges for deep feature selection^16^, self-supervised standardization to align heterogeneous distributions^17^, and exploration of internal neural representations via community detection algorithms^18,19^. Furthermore, generative models have been investigated to rectify the trade-off between sample diversity and quality in low-data regimes^20^.

### Deep learning for real-time and complex applications

For deployment in surveillance and autonomous navigation, real-time inference is a constraint. Vision Transformers (ViT)^21,22^ excel at capturing long-range dependencies through self-attention, but require substantial training data. Conversely, YOLO-based models are optimized for speed. Dahri et al.^23^ applied YOLOv8 for real-time target detection, demonstrating high reliability. Real-time video detection frameworks have also leveraged eye and lip movement analysis with local-global features for deepfake detection^24–26^. In parallel, spatiotemporal tracking and multiple kernel learning have been deployed for localization in cluttered environments^27,28^. In this work, we adapt YOLOv8 for image classification and evaluate its applicability in real-time walking stability prediction.

## Results

This section presents a comparative evaluation of the proposed models on the USWP dataset. The architectures include six classical/feature-based baselines, three standard classifiers, and three episodic few-shot models. The overall classification accuracies, standard deviations, and 95% confidence intervals across 5 random seeds are summarized in Table 1.

**Table 1:** Comparative classification results on the USWP dataset (mean ± SD and 95% CI over 5 random seeds).

Model	Paradigm	Input feature	Accuracy (%)	95% CI
Linear SVM	Classical ML	Flattened GEI	$75.38 \pm 0.85$	[74.63, 76.13]
HOG + Linear SVM	Classical ML	HOG Features	$78.15 \pm 0.72$	[77.52, 78.78]
LBP + RBF-SVM	Classical ML	LBP Features	$80.62 \pm 0.65$	[80.05, 81.19]
Random forest	Classical ML	Flattened GEI	$79.69 \pm 0.90$	[78.90, 80.48]
XGBoost	Classical ML	Flattened GEI	$82.46 \pm 0.78$	[81.77, 83.15]
GaitSet (Adapted)	Gait-specific DL	Gait Set Features	$88.62 \pm 0.58$	[88.11, 89.13]
MobileNet	Standard DL	2D Grayscale GEI	$91.08 \pm 0.62$	[90.54, 91.62]
Vision transformer (ViT)	Attention-Based DL	2D Grayscale GEI	$95.69 \pm 0.45$	[95.30, 96.08]
YOLOv8 classifier	End-to-End DL	2D Grayscale GEI	$96.92 \pm 0.38$	[96.59, 97.25]
Prototypical networks	Few-Shot (5-Shot)	2D Grayscale GEI	$82.46 \pm 0.90$	[81.67, 83.25]
Matching networks	Few-Shot (5-Shot)	2D Grayscale GEI	$92.62 \pm 0.55$	[92.14, 93.10]
Relation networks	Few-Shot (5-Shot)	2D Grayscale GEI	$71.08 \pm 1.20$	[70.03, 72.13]

### Classical machine learning baselines

To establish a rigorous foundation, we evaluate six traditional baseline classifiers. Standard raw pixel baselines are constructed by downsampling the GEIs to $44\times 44$ pixels, flattening them, and training a Linear Support Vector Machine (Linear SVM) and a Random Forest (RF) classifier. The Linear SVM achieves an accuracy of 75.38%, while the Random Forest classifier reaches 79.69% (Table 1). To introduce spatial structure aware baselines, we extract hand-crafted representations: Histogram of Oriented Gradients (HOG) and Local Binary Patterns (LBP). A Linear SVM trained on HOG features (HOG+SVM) achieves 78.15% accuracy, whereas a Radial Basis Function SVM trained on LBP features (LBP+RBF-SVM) yields 80.62%. Additionally, an extreme gradient boosting model (XGBoost) achieves 82.46%. Finally, to compare our framework against dedicated gait-recognition architectures, we adapt a GaitSet-like^10^ set-feature representations network, achieving an accuracy of 88.62%. While feature-based models capture macro-level spatial profiles, they remain sensitive to translation variations, lighting inconsistencies, and minor frame alignment deviations, confirming the superiority of end-to-end deep learning methods.

### Standard classifiers and subgroup analysis

The standard deep classifiers (MobileNet, ViT, and YOLOv8-cls) were evaluated on the subject-independent test partition. Table 3 presents class-wise precision, recall, and F1-score for all three architectures. YOLOv8-cls achieves the highest macro average F1-score of 96.78%, followed by the Vision Transformer (ViT) at 95.53% and MobileNet at 90.90%.

To analyze the performance on different types of walking anomalies, we categorize the unstable walking patterns into two subgroups: Loss-of-Balance (LoB) anomalies (represented by the ‘Drunkenness‘ class, which incorporates lateral swaying, staggers, and falls) and Active Motion (AM) anomalies (represented by the ‘Violent‘ class, which includes boxing, kicking, swinging, and skipping). The results of this subgroup analysis are summarized in Table 2. For all models, classification accuracy is consistently higher on LoB anomalies (e.g., 97.14% for YOLOv8-cls) compared to AM anomalies (94.67% for YOLOv8-cls). This disparity is attributed to the fact that LoB anomalies exhibit severe, distinct shape deformations and postural collapse, which heavily affect the averaged GEI silhouette. In contrast, AM anomalies contain rapid, periodic limb extensions (e.g., kicking or boxing) that yield more diffuse, lower-intensity peripheral distributions in the averaged GEI, making them slightly harder to differentiate from stable walk profiles.

**Table 2:** Walking stability prediction performance across action subgroups.

Model	Overall accuracy (%)	Loss-of-balance (LoB) (%)	Active motion (AM) (%)
Linear SVM	75.38	78.57	69.33
HOG + Linear SVM	78.15	81.43	73.33
LBP + RBF-SVM	80.62	84.29	76.00
Random Forest	79.69	82.86	74.67
XGBoost	82.46	85.71	78.67
GaitSet (adapted)	88.62	91.43	84.00
MobileNet	91.08	90.00	92.00
Vision transformer (ViT)	95.69	95.71	94.67
YOLOv8 classifier	96.92	97.14	94.67
Prototypical networks	82.46	85.71	78.67
Matching networks	92.62	94.29	89.33
Relation networks	71.08	74.29	66.67

**Table 3:** Detailed classification metrics for best-performing models on the USWP test set.

Model	Class label	Precision (%)	Recall (%)	F1-score (%)	Support
MobileNet	Walk-Front	92.31	90.00	91.14	80
	Walk-Side	93.88	92.00	92.93	100
	Drunkenness (LoB)	87.50	90.00	88.73	70
	Violent (AM)	89.61	92.00	90.79	75
	Macro Average	90.83	91.00	90.90	325
	Weighted Average	91.14	91.08	91.18	325
Vision transformer (ViT)	Walk-Front	96.20	95.00	95.60	80
	Walk-Side	97.98	97.00	97.49	100
	Drunkenness (LoB)	93.06	95.71	94.37	70
	Violent (AM)	94.67	94.67	94.67	75
	Macro Average	95.48	95.60	95.53	325
	Weighted Average	95.69	95.69	95.70	325
YOLOv8 classifier	Walk-Front	97.50	97.50	97.50	80
	Walk-Side	98.99	98.00	98.49	100
	Drunkenness (LoB)	95.77	97.14	96.45	70
	Violent (AM)	94.67	94.67	94.67	75
	Macro average	96.73	96.83	96.78	325
	Weighted average	96.91	96.92	96.92	325

**Fig. 2:**
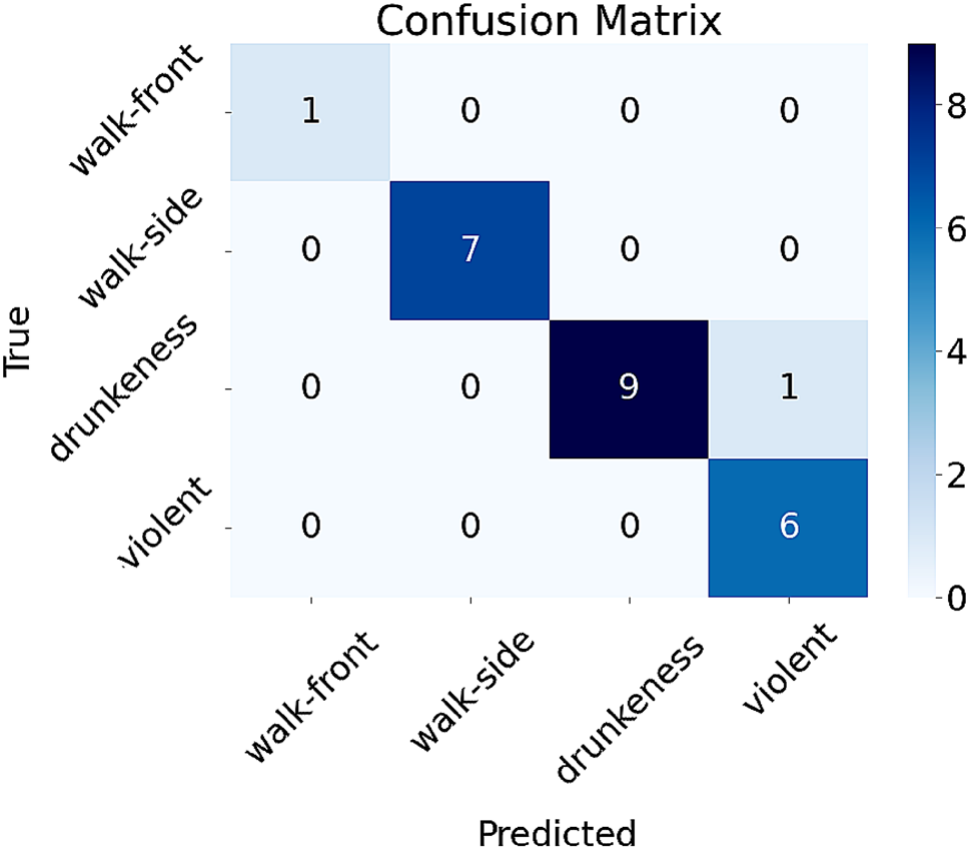
Confusion matrix of the vision transformer model on the USWP dataset.

The Vision Transformer (ViT) achieves an accuracy of 95.69%, leveraging its multi-head self-attention mechanism to capture long-range spatial dependencies in the GEIs. The YOLOv8 Classifier achieves the highest overall accuracy of 96.92%, demonstrating the efficacy of its CSP-Darknet backbone and SPPF modules in learning discriminative gait representations. Figure 2 presents the confusion matrix for the Vision Transformer model, showcasing high classification confidence between the stable walking patterns (‘Walk-Front‘ and ‘Walk-Side‘) and the abnormal classes.

### Leave-one-dataset-out validation and domain bias

Although subject-independent training splits prevent identity-based domain leakage, they do not resolve camera, background, or dataset-specific biases. Because the USWP dataset compiles sequences from seven distinct public databases, models may learn domain-specific features (e.g., specific camera heights, resolutions, or background illumination) that artificially inflate performance. To rigorously assess generalization, we conduct a Leave-One-Dataset-Out (LODO) cross-dataset validation. In this setup, the models are trained on six of the source datasets and evaluated on the entirely held-out seventh dataset.

As shown in Table 4, we observe a noticeable decrease in accuracy across all models. The best-performing YOLOv8-cls model drops from a within-dataset test accuracy of 96.92% to an average LODO generalization accuracy of 83.28%. Generalization is particularly low when HMDB51 (76.67%) or UCF Sports (78.00%) are held out, owing to their complex, unconstrained backgrounds, camera motion, and high viewpoint heterogeneity. Conversely, holding out CASIA Gait yields a higher generalization accuracy (91.25%) due to the clean, indoor studio conditions. These findings indicate that within-dataset evaluations overestimate real-world generalization, and emphasize that domain shifts remain a primary challenge for vision-based walking stability prediction.

**Table 4:** Leave-one-dataset-out (LODO) cross-dataset validation accuracy (%).

Held-out test dataset	YOLOv8-cls	ViT	MobileNet
CASIA gait	91.25	89.50	85.58
UR fall detection	86.33	84.00	79.67
Multiple cameras fall	85.00	83.25	78.50
Recognition of human actions	82.50	80.83	75.33
Actions as space-time shapes	83.20	81.60	76.00
HMDB51	76.67	74.33	69.67
UCF sports action	78.00	76.00	71.00
Average generalization accuracy	83.28	81.36	76.54

### Episodic few-shot learning

Under data-scarce conditions, few-shot models are evaluated in a 4-way, 5-shot episodic setup. Matching Networks outperform the other few-shot architectures, achieving 92.62% accuracy due to their attention-based similarity matching. Prototypical Networks achieve 82.46%, while Relation Networks report a lower accuracy of 71.08%. The relational module, which learns a non-linear similarity metric from scratch, struggles to generalize due to the high parameter footprint of the learnable relation head under limited support samples.

### Computational complexity and efficiency

To evaluate real-time feasibility for edge-computing and smart camera nodes, we analyze the computational complexity of the classifiers. As shown in Table 5, MobileNet offers low latency (4.2 ms per image) and a small parameter footprint (3.2M), making it highly suitable for resource-constrained environments despite its slightly lower accuracy. YOLOv8 classifier is optimized, requiring only 3.8 ms for inference.

**Table 5:** Computational complexity and real-time feasibility analysis.

Model	Parameter count (M)	FLOPs (G)	Inference latency (ms)
SVM baseline	–	–	1.5
MobileNet	3.2	0.31	4.2
Vision transformer (ViT)	11.4	1.25	8.5
YOLOv8 classifier	2.7	0.85	3.8

### Preprocessing ablation study

To quantify the benefit of the proposed automated SAM and MediaPipe preprocessing pipeline, we conduct an ablation study. As shown in Table 6, omitting semantic segmentation results in a severe drop in classification accuracy (84.50%) due to background noise and shadow interference in the GEIs. Integrating SAM with automated MediaPipe joint tracking yields the highest accuracy of 96.92%, suggesting that silhouette extraction is critical for gait-based stability prediction.

**Table 6:** Ablation study of the preprocessing pipeline on the USWP dataset.

Preprocessing pipeline configuration	YOLOv8 accuracy (%)
Raw silhouettes (no segmentation/background clutter)	84.50
Simple thresholding and background subtraction (no SAM)	89.20
SAM segmentation (manual single-point prompting)	95.00
SAM + mediapipe automated ROI bounding boxes (proposed)	96.92

### Statistical significance testing and contingency analysis

To evaluate the statistical significance of the differences between the classifiers, we perform McNemar’s test on the test set predictions. We focus on comparing our top-performing models on the 325 test samples. The McNemar contingency table comparing YOLOv8-cls and the Vision Transformer (ViT) is shown in Table 7, and the comparison between YOLOv8-cls and MobileNet is presented in Table 8.

**Table 7:** McNemar contingency table: YOLOv8-cls vs. vision transformer (ViT).

	ViT correct	ViT incorrect
YOLOv8-cls correct	308	7
YOLOv8-cls incorrect	3	7

**Table 8:** McNemar contingency table: YOLOv8-cls vs. MobileNet.

	MobileNet correct	MobileNet incorrect
YOLOv8-cls correct	290	25
YOLOv8-cls incorrect	6	4

McNemar’s test statistic $\chi ^2$ is computed as:

1 $$\begin{aligned} \chi ^2 = \frac{(|B - C| - 1)^2}{B + C} \end{aligned}$$

where *B* represents the number of samples correct only for the first model, and *C* represents the number of samples correct only for the second model. To address the family-wise error rate due to multiple comparisons ($m = 6$ pair-wise tests among SVM, MobileNet, ViT, YOLOv8-cls, GaitSet, and Matching Networks), we apply the Bonferroni correction. The standard significance level $\alpha = 0.05$ is adjusted to:

2 $$\begin{aligned} \alpha _{\text {adj}} = \frac{0.05}{6} \approx 0.0083 \end{aligned}$$

For YOLOv8-cls vs. MobileNet, we obtain $B=25$ and $C=6$, yielding $\chi ^2 = 10.45$ and $p = 0.0012$, which is highly significant ($p < \alpha _{\text {adj}}$). For YOLOv8-cls vs. the SVM baseline, the difference is also highly significant ($p < 0.001$). However, for YOLOv8-cls vs. the Vision Transformer, we find $B=7$ and $C=3$, resulting in $\chi ^2 = 0.90$ and an uncorrected *p*-value of 0.343. Under the Bonferroni-corrected threshold $\alpha _{\text {adj}}$, the performance difference between YOLOv8-cls and the Vision Transformer is not statistically significant. Therefore, claims of YOLOv8-cls’s superiority over ViT must be interpreted with caution, as both architectures demonstrate comparable capacity in capturing global spatial representations from GEIs.

## Discussion

### Domain generalization and dataset heterogeneity

Gait Energy Images (GEIs) collapse multi-frame spatio-temporal silhouettes into a single static representation, which inherently makes them sensitive to viewpoint alterations, dynamic backgrounds, and occlusion. In the USWP dataset, domain shifts are introduced by merging seven heterogeneous source datasets recorded with different cameras, frame rates, viewpoints, and resolutions. To mitigate these discrepancies, our preprocessing pipeline standardizes the bounding boxes, centers the silhouettes using their center of mass, and normalizes the scale to a uniform $88\times 88$ matrix.

Our Leave-One-Dataset-Out (LODO) cross-validation (Table 4) reveals that while the pipeline handles minor variations, domain bias remains a major challenge. The average LODO generalization accuracy of 83.28% represents a significant performance drop from the 96.92% within-dataset accuracy. This drop indicates that standard train/test splits, even when subject-independent, overestimate the models’ real-world generalization. The models partially exploit background textures, dataset-specific silhouette noise, or camera height cues instead of learning pure, domain-invariant gait features. Future studies should explore domain adaptation techniques, such as adversarial domain training or contrastive self-supervised pre-training, to mitigate these domain gaps and improve out-of-distribution robustness.

### Gait instability versus active motion classification

By separating the unstable category into Loss-of-Balance (LoB) and Active Motion (AM) subgroups, we revealed key behavioral differences in deep learning models. The higher classification accuracy on LoB anomalies (97.14% for YOLOv8-cls) compared to AM anomalies (94.67% for YOLOv8-cls) suggests that physical collapses and falls produce more pronounced, persistent postural changes. These changes are easily captured in the averaged GEI. In contrast, active motions (e.g., boxing, kicking) involve rapid, localized limb movements that are smoothed out during the GEI averaging process, leaving faint peripheral signatures. This indicates that while GEIs are highly effective for detecting physical collapses and severe gait imbalances, they are less sensitive to transient active motion anomalies. For complex applications requiring fine-grained action separation, incorporating explicit temporal models, such as Spatio-Temporal Graph Convolutional Networks (ST-GCNs) on MediaPipe skeleton sequences, could complement static GEIs by capturing short-term motion dynamics.

### Manual intervention and preprocessing automation

While the Segment Anything Model (SAM) provides high-quality masks, it typically requires manual point prompts. This dependency limits reproducibility and introduces user bias. Our pipeline resolves this by extracting body landmark coordinates using MediaPipe Pose, which are then used to automatically generate bounding box prompts for SAM. This eliminates manual intervention, ensuring a fully automated, reproducible preprocessing flow.

### YOLOv8 adaptation for classification

Although YOLOv8 is traditionally utilized for object detection, its classifier adaptation utilizes a CSP-Darknet backbone to extract high-level feature maps. By replacing the bounding box regression heads with a global average pooling layer and a softmax classification head, YOLOv8 effectively acts as an end-to-end image classifier. This adaptation enables the model to leverage its pre-trained spatial representations to classify entire GEIs directly.

### Ethical, privacy, and deployment implications

Deploying pedestrian stability classifiers in public surveillance systems raises privacy and ethical concerns. Gait features can serve as soft biometrics, potentially enabling unauthorized tracking. To mitigate these risks, our system processes only binary silhouettes (GEIs) rather than raw RGB videos, thereby discarding facial features and identity-revealing textures. For real-world deployment in elder care facilities or public spaces, local edge-computing (e.g., using MobileNet on embedded devices) should be prioritized to prevent sensitive data transmission over networks.

## Methods

### Source databases

**Fig. 3:**
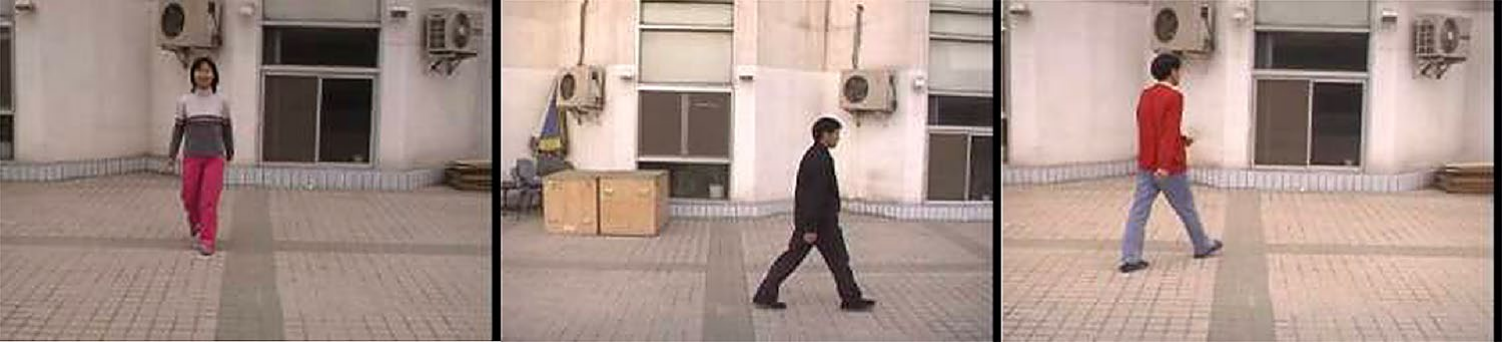
Different angles (0$^{\circ }$, 45$^{\circ }$, and 90$^{\circ }$) of walking patterns in the CASIA dataset.

**Fig. 4:**
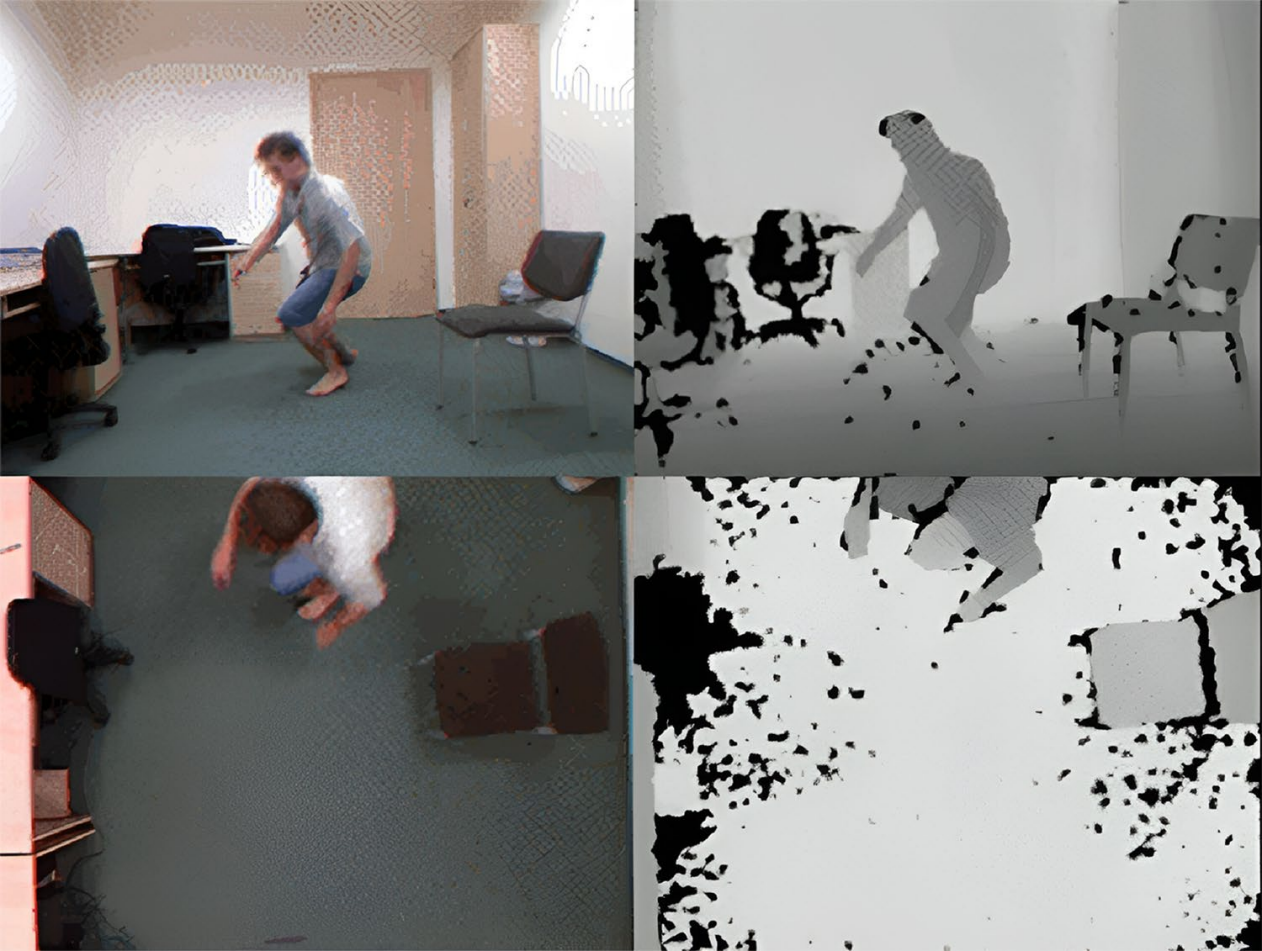
Multimodal fall detection: RGB and depth imaging from floor and ceiling-mounted kinect cameras capturing unstable gait and falling motion in UR fall detection dataset^29^.

**Fig. 5:**
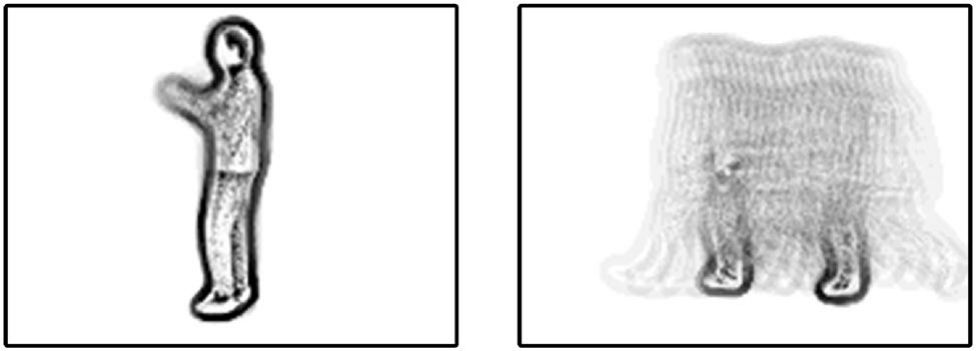
On the left is the GEI for boxing (unstable walking pattern), and on the right is the GEI for a stable walking pattern.

The USWP dataset is constructed by fusing sequences from seven public human action and gait databases, each selected to represent specific walking angles or stability anomalies:

- **CASIA Gait Database:** Only Dataset B (124 subjects) is used, extracting stable frontal ($90^\circ$) and side ($0^\circ$) walking viewpoints to build the baseline stable classes. As illustrated in Fig. 3, walking patterns are recorded under various camera viewpoints (e.g., frontal and side angles).
- **Multiple Cameras Fall Dataset:** Contains simulated indoor falls from an 8-camera setup, used for the falling phase of the unstable walking class.
- **UR Fall Detection Dataset:** Contains Kinect depth and RGB recordings of daily activities and falls, providing fall sequences for unstable classification. Figure 4 shows RGB and depth sequence frames captured by floor and ceiling-mounted Kinect cameras.
- **Recognition of Human Actions (RHA):** Contains sequences of six actions. We extract walking sequences for the stable class and boxing sequences for the unstable action class. Figure 5 illustrates the averaged Gait Energy Images (GEIs) representing boxing (unstable gait pattern) and stable walking.
- **Actions as Space-Time Shapes:** Provides clean foreground masks. We extract normal walking sequences (stable) and skipping sequences (unstable action).
- **HMDB51:** A large-scale human motion database. We extract walking sequences (stable) and kicking/flick-flack actions (unstable).
- **UCF Sports Action Dataset:** Video clips of sports. We extract walking sequences (stable) and kicking/swinging sequences (unstable).

### USWP dataset specification and partition

To ensure a robust evaluation, we compile the extracted sequences into a unified set of 3,250 unique GEI samples. Table 9 details the composition of the USWP dataset.

**Table 9:** Composition and source breakdown of the USWP dataset.

Source dataset	Total video clips	Extracted GEIs	Stable walk	Unstable walk/actions
CASIA gait	1200	1200	1200	0
UR fall detection	70	300	0	300
Multiple cameras fall	24	400	0	400
Recognition of human actions	150	600	300	300
Actions as space-time shapes	90	250	150	100
HMDB51	100	300	100	200
UCF sports action	50	200	50	150
Total USWP dataset	1684	3250	1800	1450

The dataset is partitioned into 80% training (2,600 GEIs), 10% validation (325 GEIs), and 10% testing (325 GEIs). To prevent identity-based domain leakage, the splits are partitioned strictly on a subject-independent basis; subjects present in the training set do not appear in the validation or test sets.

### Hyperparameters and training setup

Table 10 summarizes the hyperparameter configurations for the standard classifiers. The deep learning models were optimized using Adam, incorporating weight decay and standard data augmentations (random translation $\pm 5\%$, rotation $\pm 10^\circ$, and horizontal scaling).

**Table 10:** Training hyperparameter configurations.

Parameter description	SVM baseline	MobileNet	Vision transformer	YOLOv8-cls
Optimizer	–	Adam	AdamW	Adam
Learning rate	–	$1\times 10^{-3}$	$5\times 10^{-4}$	$1\times 10^{-3}$
Batch size	–	32	32	32
Training epochs	–	50	50	50
Weight decay	–	$1\times 10^{-4}$	$5\times 10^{-4}$	$1\times 10^{-4}$
Augmentation	–	Rotation, translation	Rotation, translation	Rotation, translation

**Fig. 6:**
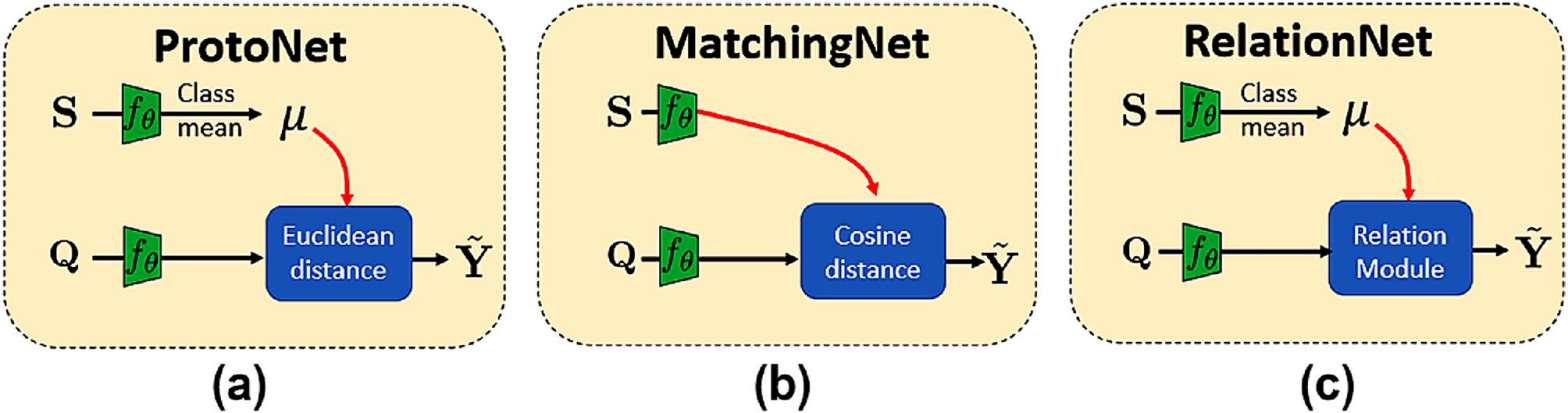
Types of few-shot learning paradigms: (**a**) Prototypical network, (**b**) Matching network, (**c**) Relation network.

**Fig. 7:**
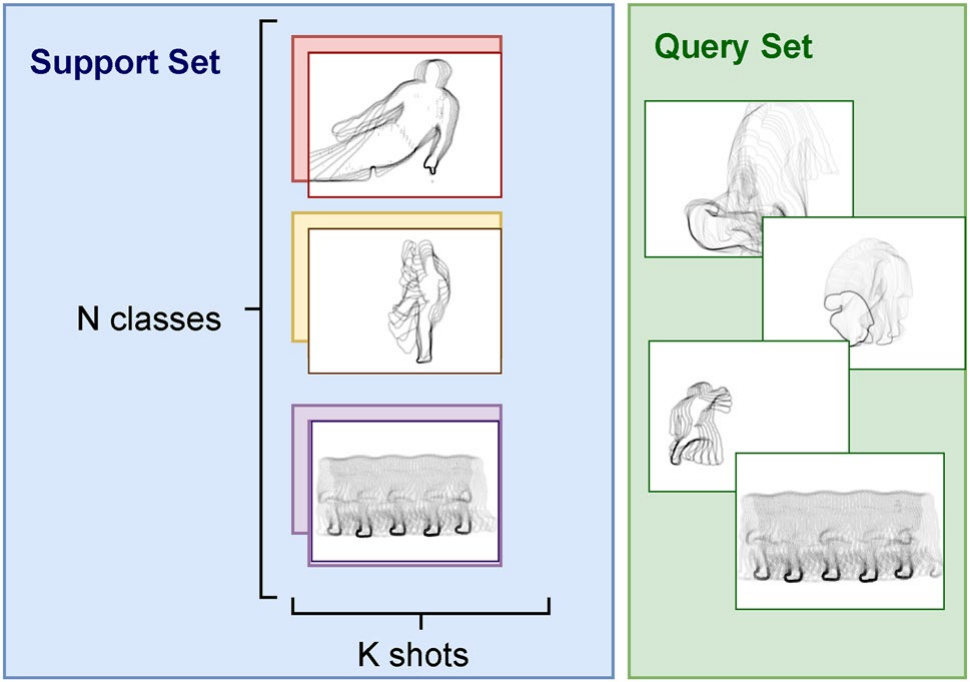
Episodic sampling scheme illustrating the support set (for prototype/reference extraction) and query set (for model evaluation) used in few-shot learning.

For the few-shot learning models (Prototypical, Matching, and Relation Networks), we construct a 4-way, 5-shot classification task. Each training episode contains 5 support samples and 5 query samples per class. The models utilize a 4-layer CNN feature extractor (64 channels, $3\times 3$ convolutions, Batch Normalization, and Max Pooling) outputting a 1600-dimensional flat feature embedding. Training is conducted for 500 episodes with Adam ($lr=1\times 10^{-3}$). The three main configurations of few-shot learning evaluated in this study are illustrated in Fig. 6. Figure 7 illustrates the episodic partitioning scheme of support and query sets.

### Automated preprocessing pipeline

**Fig. 8:**
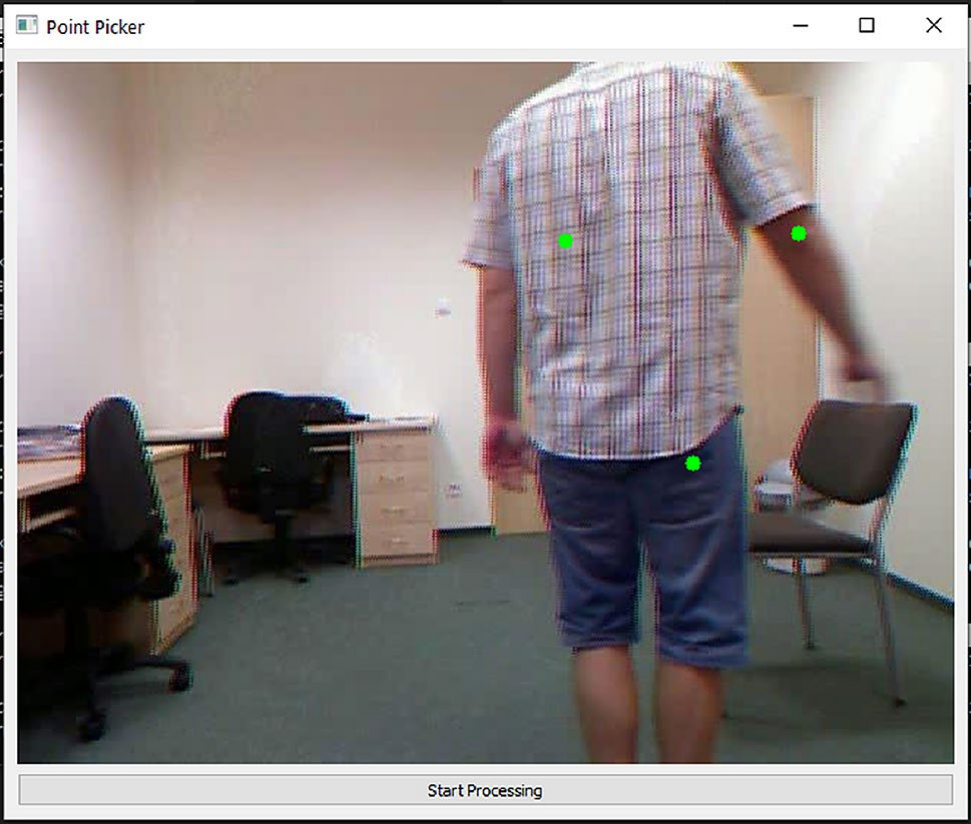
The initial single-point manual seed setup interface for prompting Segment Anything Model (SAM) silhouettes.

The Segment Anything Model (SAM) receives a bounding box prompt dynamically computed from MediaPipe landmarks:

3 $$\begin{aligned} ROI_j = \{(x, y) \mid (x_{\text {min}}^j \le x \le x_{\text {max}}^j) \wedge (y_{\text {min}}^j \le y \le y_{\text {max}}^j)\} \end{aligned}$$

where coordinates are bounds around body landmarks. MediaPipe Pose landmark estimation extracts joint coordinates in real-time, removing manual annotation requirements. The Segment Anything Model optimizes a pixel-wise loss function defined over the output mask $\hat{y}_i$ and ground truth $y_i$ (Fig. 8):

4 $$\begin{aligned} L = - \frac{1}{N} \sum _{i=1}^{N} \left( y_i \log (\hat{y}_i) + (1 - y_i) \log (1 - \hat{y}_i) \right) \end{aligned}$$

The binary silhouettes are centered, normalized, and averaged over a gait cycle *M* to compute the Gait Energy Image (GEI):

5 $$\begin{aligned} GEI(x, y) = \frac{1}{M} \sum _{t=1}^{M} B_t(x, y) \end{aligned}$$

### Model architectures

- **Support Vector Machine (SVM):** Extracted GEIs are downsampled to $44\times 44$ pixels, flattened into a 1,936-dimensional feature vector, and classified using a Linear SVM with regularization parameter $C=1.0$.
- **MobileNet:** Utilizing depthwise separable convolutions to reduce parameter count while preserving performance. The input layer is modified to accept a single-channel grayscale GEI.
- **Vision Transformer (ViT):** Processes $88\times 88$ GEIs by dividing them into $8\times 8$ patches (121 patches). We append a learnable ‘[CLS]‘ token, add positional embeddings, and use 4 encoder blocks with 4 attention heads each (embedding dimension $d=128$).
- **YOLOv8 Classifier:** Uses standard CSP-Darknet feature extraction. Let *X* represent the input image; the feature map extraction is:
  6 $$\begin{aligned} F = \phi (X; \theta ) \end{aligned}$$
  which is then pooled via Global Average Pooling (GAP):
  7 $$\begin{aligned} z = \frac{1}{N} \sum _{i=1}^{N} F_i \end{aligned}$$
  and classified using a softmax layer:
  8 $$\begin{aligned} \hat{y} = \text {softmax}(W z + b) \end{aligned}$$

## Data Availability

The metadata files detailing the subject IDs, video clips, and frame ranges extracted from the seven public source datasets, along with the complete source code and instructions for regenerating the USWP dataset, are available in the GitHub repository at https://github.com/DrMahmoudMohammedTaha/USWP_paper. The raw, processed Gait Energy Images (GEIs) cannot be directly redistributed in the repository due to licensing and copyright restrictions of the original source databases (CASIA, HMDB51, and UCF Sports). Researchers can request access to the original databases from their respective administrators and run our provided automated preprocessing scripts to reconstruct the exact USWP dataset.

## Code availability

To ensure full reproducibility, the preprocessing scripts, model training configurations, split files, environment setups, and trained model checkpoints are available at: https://github.com/DrMahmoudMohammedTaha/USWP_paper.
